# Association between dietary patterns and different obesity phenotypes among Inner Mongolia adults: a cross-sectional study

**DOI:** 10.3389/fnut.2025.1660337

**Published:** 2025-09-02

**Authors:** Xin Liu, Pengpu Wang, Xinyan Wang, Ke Han, Huiqiu Zheng, Jing Zhao, Qianqian Du, Bowen Zhou, Bowen Wu, Xuemei Wang

**Affiliations:** ^1^School of Public Health, Inner Mongolia Medical University, Hohhot, China; ^2^Ordos Center for Disease Control and Prevention, Ordos, China

**Keywords:** obesity, metabolic abnormalities, obesity phenotypes, dietary patterns, Chinese Dietary Balance Index-22

## Abstract

**Background:**

This study aimed to investigate the association between dietary patterns and obesity phenotypes among adults in Inner Mongolia using the Chinese Dietary Balance Index (DBI-22).

**Methods:**

A cross-sectional study was conducted among adults in Ordos, Inner Mongolia. Sociodemographic information, lifestyle, and physical activity were collected using a comprehensive questionnaire. Dietary data were collected with a validated semi-quantitative food frequency questionnaire (FFQ) to assess participants’ dietary intake over the past year. Body mass index (BMI) and metabolic status were measured through physical examinations and biochemical tests. Principal component analysis (PCA) was used to identify dietary patterns. A generalized linear model was applied to assess the association between the DBI-22 and dietary patterns. Multinomial logistic regression was performed to investigate the association between dietary patterns and obesity phenotypes.

**Results:**

Three dietary patterns were identified in Mongolia: the plant-based dietary pattern, the northern pastoral dietary pattern, and the northern traditional dietary pattern. Using metabolically healthy non-obesity (MHNO) as the reference group, higher adherence to the northern pastoral dietary pattern increased the risk of metabolically healthy obesity (MHO, OR 1.44, 95% CI 1.0, 2.08) but decreased the risk of metabolically unhealthy non-obesity (MUNO, OR 0.70, 95% CI 0.54, 0.91). Higher adherence to the plant-based dietary pattern was associated with a reduced risk of MHO (OR 0.65, 95% CI 0.47, 0.91). Higher adherence to the northern traditional dietary pattern was linked to an elevated risk of metabolically unhealthy obesity (MUO, OR 1.57, 95% CI 1.2, 2.06). Generalized linear models showed that the plant-based dietary pattern and the northern pastoral dietary pattern demonstrated relatively balanced nutritional characteristics, while the northern traditional dietary pattern was characterized by relative imbalance.

**Conclusion:**

Adherence to the northern pastoral dietary pattern increased the risk of MHO and reduced the risk of MUNO, while adherence to the plant-based dietary pattern reduced the risk of MHO. Both patterns demonstrated balanced diet quality. In contrast, adherence to the nutritionally imbalanced northern traditional dietary pattern increased the risk of MUO, highlighting the need for nutritional intervention.

## 1 Introduction

Obesity has emerged as the most pressing global public health challenge (1). Recent data indicate that the obesity prevalence rate among Chinese adults has reached 16.4% (2). Obesity is currently ranked as the sixth most significant contributor to both mortality and disability in China (3). Obesity frequently coexists with other metabolic abnormalities (such as hypertension, diabetes, and dyslipidemia), interacts synergistically, and jointly contributes to the pathological development of atherosclerosis, thereby serving as critical risk factors for cardiovascular diseases (4). A meta-analysis of prospective cohort studies has consistently shown a strong association between body mass index (BMI) and adverse health outcomes. Nevertheless, individuals with comparable BMI values often exhibit substantial variability in the prevalence of obesity-related metabolic abnormalities (5), and the risk of obesity-related diseases also differs (6). In the previous research, participants were frequently categorized as metabolically healthy or unhealthy based on the presence or absence of metabolic abnormalities (7). Metabolic abnormalities were defined according to the adult Panel III (ATP III) criteria (8). Obese individuals with a metabolically unhealthy status may have a worse prognosis and a higher risk of mortality compared to those with a metabolically healthy status (9). Furthermore, in the metabolically unhealthy obesity (MUO) population, the incidence of cardiovascular disease increased by 1.63 times and the cardiovascular mortality rate increased by 1.77 times, whereas the incidence of diabetes increased by 3.49 times (10). Therefore, the obesity phenotype based on metabolic status and BMI provides a more comprehensive framework for assessing the health risks associated with obesity and designing appropriate intervention programs to manage obesity and its adverse consequences (11).

Dietary factors are a primary treatment approach for managing obesity and metabolic abnormalities (12). Dietary patterns provide a more comprehensive assessment of the overall diet compared to single nutrients or foods and better reflect the true relationship between diet and disease. This approach makes them particularly favorable to clinical and public dietary advice (13). Numerous studies have examined the association between dietary patterns and both obesity and metabolic abnormalities (14). Evidence suggests that Western dietary patterns often lead to weight gain and insulin resistance, while the Mediterranean diet has also been proven effective in improving metabolic health and reducing obesity risk (15). The Dietary Approaches to Stopping Hypertension (DASH) dietary pattern not only helps to control hypertension but also improves the weight, blood lipids, insulin sensitivity, and blood glucose levels of patients with metabolic syndrome (16). The Chinese Dietary Guidelines highlight that the Eastern dietary pattern reduces the risk of both obesity and metabolic abnormalities.

However, Inner Mongolia, located in northern China, has developed a distinctive dietary pattern due to its unique geographical factors, climate factors, and dietary habits (17). The prevalence of obesity and metabolic abnormalities in this region is relatively high and is a major public health issue (18, 19). Therefore, this study aimed to investigate the association between dietary patterns and obesity phenotypes among adults in Inner Mongolia. The Chinese Dietary Balance Index-22 (DBI-22) scores were used to evaluate the quality of the dietary patterns. Our findings provided a novel strategy for the prevention of obesity and related diseases.

## 2 Methods

### 2.1 Study design

Participants were recruited from adults (≥18 years) enrolled in the Ordos Resident Health White Paper Project Program. A cross-sectional study was conducted to investigate sociodemographic, lifestyle, and dietary data using a multi-stage stratified random sampling method. The exclusion criteria included individuals >79 years (*n* = 12); participants without complete dietary survey data (*n* = 9); participants with non-biological blood samples (*n* = 113); participants with extreme energy intake (<800 or >4,200 kcal/day for men, <600 or >3,500 kcal/day for women, *n* = 43); those without physical activity measurements (*n* = 202); those with missing data (*n* = 40); and those who were underweight (BMI < 18.5 kg/m^2^, *n* = 57). Therefore, a total of 3,672 participants were included in the present analysis. This project was approved by the Biomedical Research Ethics Committee of Inner Mongolia Medical University (No. YKD202301132). All participants signed the written informed consent form before the investigation. Height and weight measurements of participants were obtained directly by trained investigators, with concurrent collection of blood samples. The laboratory director supervised the quality control process for the samples at the on-site laboratory.

### 2.2 Dietary data collection

Dietary intake information was collected by a semi-quantitative food frequency questionnaire (FFQ), which was a modified and adapted FFQ based on the China Adult Chronic Diseases and Nutrition Surveillance (2015) (20). To accurately capture the dietary habits of adults in Ordos, Inner Mongolia, some local specialty foods, such as naked oat flour, buckwheat flour, milk tofu, butter, and boiled milk tea, were added to this adapted FFQ. A pre-survey was conducted to assess the reliability and validity of the questionnaire (21). The validated FFQ consisted of 12 categories: staple foods, beans, vegetables, fungi and algae, fruits, dairy products, meats, aquatic products, eggs, beverages, alcohol, and others. Participants were required to report daily, weekly, monthly, and annual food consumption frequencies through face-to-face interviews conducted by uniformly trained investigators. Food consumption was converted to per capita daily intakes to assess participants’ dietary intake over the past year. The questionnaire collected information regarding the frequency and type of alcohol consumption (high liquor, low liquor, beer, rice wine, wine, and other fruit wines) and average alcohol consumption. Average daily alcohol intake was calculated based on the guidelines outlined in the Chinese Chronic Disease and Nutrition Surveillance Survey Manual (22). Research subjects were interviewed face-to-face by uniformly trained investigators.

### 2.3 Chinese Dietary Balance Index-22

The Chinese Dietary Balance Index (DBI-22) is a tool for assessing dietary quality, developed in accordance with the “Chinese Dietary Guidelines” (22). The DBI-22 includes three indicators of dietary quality: the higher-bound score (HBS), the lower-bound score (LBS), and the diet quality distance (DQD). The HBS is obtained by summing all positive scores, serving as an indicator of excessive food consumption. The LBS is determined by summing the absolute values of all negative scores, indicating insufficient food intake. The DQD reflects dietary imbalance and is calculated by summing the absolute values of both positive and negative scores. The possible score ranges for the HBS, LBS, and DQD are 0–44, 0–60, and 0–96, respectively (23).

### 2.4 Definition of obesity phenotypes

Based on the guidelines for the prevention and control of overweight and obesity among Chinese adults, BMI was categorized as non-obesity (18.5 ≤ BMI < 28 kg/m^2^) and obesity (BMI ≥ 28 kg/m^2^) (24). Metabolic abnormalities were assessed using the Adult Treatment Group III (ATP-III) criteria (25), and participants meeting two or more of the following four criteria were classified as having metabolic abnormalities: high triglycerides (≥ 1.7 mmol/L), elevated systolic blood pressure (≥ 130 mmHg) or diastolic blood pressure (≥ 85 mmHg), high fasting blood glucose (≥ 5.6 mmol/L), and low high-density lipoprotein cholesterol (1.04 mmol/L in men and 1.29 mmol/L in women). Due to statistical collinearity with BMI, the waist circumference index was excluded. Participants were categorized into four groups based on their BMI and metabolic status: metabolically healthy non-obesity (MHNO); metabolically healthy obesity (MHO); metabolically unhealthy non-obesity (MUNO), and metabolically unhealthy obesity (MUO).

### 2.5 Definition of other variables

Age was considered a continuous variable. Ethnic groups were classified into Han, Mongolian, and other minorities—the latter including all ethnic groups residing in Inner Mongolia other than Han and Mongolian. Regions were classified as urban, rural, and pastoral areas according to household registration. Educational attainment was grouped into three levels: low (primary school and below), medium (secondary school or technical secondary school), and high (college level and above). Marital status was categorized as married, unmarried, and other (e.g., divorced and widowed). Smoking status was classified into three categories: non-smoker (never smoked), current smoker (smoked at least one cigarette per day for more than 1 year and currently smokes), and ex-smoker (formerly smoked but has quit). Alcohol intake was measured in units of daily alcohol consumption, and alcohol consumption was determined based on participants’ drinking behavior over the past month. Participants were categorized based on their average daily alcohol intake: for men, as non-drinkers (0 g/d), moderate drinkers (25 g/d), and heavy drinkers (>25 g/d); for women, as non-drinkers (0 g/d), moderate drinkers (15 g/d), and heavy drinkers (>15 g/d) (26). Physical activity was evaluated using the International Physical Activity Questionnaire (IPAQ), which categorized activity levels as low, moderate, and high (27). Sleep data were collected based on participants’ self-reported nightly sleep duration over the past month (28). Using these data, we categorized night-time sleep duration for analysis according to the adult sleep recommendations outlined in the American Academy of Sleep Medicine and the Sleep Research Society: <7 h (insufficient sleep), 7–9 h (adequate sleep), and >9 h (excessive sleep) (29).

### 2.6 Statistical analysis

Normally distributed continuous variables were expressed as means ± standard deviations. Categorical variables were expressed as frequencies (percentages). Analysis of variance was used to assess continuous variables. The chi-square test was used to evaluate the association between categorical variables. Multiple comparisons were adjusted using the Bonferroni Correction method (30).

Principal component analysis (PCA) was conducted to derive dietary patterns from 21 predefined food groups. Factor loadings were extracted using the varimax orthogonal rotation method. Each dietary pattern was labeled based on the food groups with factor loadings ≥ 0.30. The number of dietary patterns was determined based on the screen plot, the interpretability of food group combinations, and the proportion of explained variance. Dietary patterns were labeled according to their compositional characteristics and predominant food groups. After determining the dietary pattern, multiple regression was used to calculate factor scores for each pattern based on factor loadings and standardized daily intake of various foods. These factor scores for each dietary pattern were then divided into quartiles (Q1 to Q4) in ascending order. Higher factor scores indicated greater adherence to the dietary pattern, while lower scores indicated greater deviation from it.

The association between dietary patterns and obesity phenotypes was assessed using multinomial logistic regression, with MHNO serving as the reference group. The first quantile of the dietary pattern score was used as the reference group. Additionally, we adjusted for potential confounding factors: age, gender, region, ethnicity, marital status, education, smoking status, and physical activity. The median score of each quartile was used as a continuous variable in the regression model to test the linear trends of these associations (31). Dietary pattern quality was evaluated using generalized linear models, with LBS, HBS, and DQD as the dependent variables and dietary patterns as the independent variables after adjusting for confounders mentioned above. Subgroup analysis used physical activity as a stratification factor to explore the potential effect modification factors. The significance level was set at *α* = 0.05, and a *p*-value of < 0.05 was considered statistically significant. All statistical analyses were performed using SPSS (version 26.0; IBM Corp., Armonk, NY, United States).

## 3 Results

### 3.1 Characteristics of participants categorized according to obesity phenotypes

The MHO phenotype represented 11.5% (*n* = 421) of the overall sample, and 39.7% of the individuals were classified as obese, whereas the MHNO phenotype comprised 44.0% (*n* = 1,617) of the total population and 61.9% of those without obesity. The MUNO phenotype constituted 27.1% (*n* = 995) of the total sample and 38.1% of the non-obese subgroup, whereas the MUO phenotype made up 17.4% (*n* = 639) of the overall population and 60.3% of those classified as obese. The characteristics of the MHNO population included individuals aged 18–44 years, those residing in rural areas, those who were unmarried, those with no smoking or alcohol drinking history, and those experiencing insufficient sleep. The MHO population was characterized by residence in pastoral areas, belonging to Mongolian and other ethnic groups, having an educational level of primary school or below, having quit smoking, engaging in moderate drinking, experiencing excessive sleep, and maintaining high physical activity levels. The MUNO population was predominantly aged ≥ 45 years, urban dwelling, Han ethnicity, married, and exhibiting low physical activity. The MUO population was primarily male, divorced or widowed, with a college education level or higher, currently smoking, engaging in excessive drinking, and moderate physical activity. Among the determinants of metabolic health, except glucose, triglyceride, and total cholesterol in the MHO group, there were statistically significant differences between the other three groups and the MHNO group (*p* < 0.001) (Tables 1, 2). The average concentrations of HDL-C in the MHO, MUNO, and MUO phenotypes were all lower than those in the MHNO phenotype. Conversely, the remaining metabolic parameters in these three phenotypes were all higher than those in the MHNO phenotype (Supplementary Table S1).

**Table 1:** Demographic characteristics of different obesity phenotypes.

Characteristics	MHNO	MHO	MUNO	MUO	*p*-value
*N* (%)	1,617 (44.0)	421 (11.5)	995 (27.1)	639 (17.4)	
Age (years)	48.4 ± 12.2^a^	48.5 ± 11.4^a^	51.6 ± 11.0^b^	48.7 ± 11.1^c^	<0.001
Male (%)	860 (53.2)^a^	243 (57.7)	586 (58.9)^b^	424 (66.4)^c^	<0.001
Female (%)	757 (46.8)^a^	178 (42.3)	409 (42.1)^b^	215 (33.6)^c^	
Region group (%)					<0.001
Urban	513 (31.7)	109 (25.9)^b^	379 (38.1)^c^	215 (33.7)	
Rural	1,007 (62.3)	262 (62.2)	582 (58.5)	369 (57.8)	
Pastoral	97 (6.0)^a^	50 (11.9)^b^	34 (3.4)^c^	55 (8.6)	
Ethnic group (%)					<0.001
Han	1,367 (84.5)^a^	320 (76)^b^	901 (90.6)^c^	535 (83.7)^a^	
Mongolian and others	250 (15.5)^a^	101 (24)^b^	94 (9.5)^c^	104 (16.3)^a^	
Marital status (%)					<0.001
Unmarried	98 (6.1)^a^	19 (4.5)	22 (2.2)_b_	19 (3.0)_b_	
Married	1,464 (90.5)_a_	388 (92.2)	934 (93.9)_b_	594 (93)_b_	
Others (Divorced or Widowed)	55 (3.4)	14 (3.3)	39 (3.9)	26 (4.1)	
Education (%)					0.008
No schooling/primary school	419 (25.9)	125 (29.7)	236 (23.7)	165 (25.8)	
Middle/high school	793 (49)^a^	191 (45.4)	491 (49.4)^a^	272 (42.6)^b^	
College/university	405 (25.1)^a^	105 (24.9)	268 (26.9)	202 (31.6)^b^	

Continuous variables are presented as means ± SD, while categorical variables are presented as *n* (%). *p*-value is derived from the χ^2^ test for categorical variables and from the analysis of variance for continuous variables. Different superscript letters in the table represent a *p*-value of <0.05.

**Table 2:** Lifestyle characteristics of different obesity phenotypes.

Characteristics	MHNO	MHO	MUNO	MUO	*p*-value
Smoking Group (%)					<0.001
Never smoked	991 (61.3)^a^	244 (58)	530 (53.3)^b^	336 (52.6)^b^	
Current smoker	539 (33.3)^a^	141 (33.5)	385 (38.7)^b^	253 (39.6)^b^	
Former smoker	87 (5.4)^a^	36 (8.6)	80 (8)^b^	50 (7.8)	
Sleep Group (%)					0.394
Insufficient sleep	61 (3.8)	7 (1.7)	27 (2.7)	20 (3.1)	
Adequate sleep	1,140 (70.6)	305 (72.5)	719 (72.3)	462 (72.3)	
Excessive sleep	413 (25.6)	109 (25.9)	248 (25)	157 (24.6)	
Alcohol Group (%)					0.001
Non-drinker	1,101 (68.1)^a^	272 (64.6)	612 (61.5)^b^	392 (61.3)^b^	
Moderate drinker	345 (21.3)	105 (24.9)	235 (23.6)	145 (22.7)	
Heavy drinker	171 (10.6)^a^	44 (10.5)	148 (14.9)^b^	102 (16.0)^b^	
Physical Activity (%)					0.02
Low physical activity	1,046 (64.7)	276 (65.6)	682 (68.5)	404 (63.2)	
Moderate physical activity	102 (6.3)	19 (4.5)^b^	53 (5.3)^b^	57 (8.9)^a^	
High physical activity	469 (29)	126 (29.9)	260 (26.1)	178 (27.9)	

Categorical variables are presented as *n* (%). *p*-value is derived from the χ^2^ test. Different superscript letters in the table represent *p* < 0.05.

### 3.2 Dietary patterns

Three dietary patterns were identified through PCA: the first dietary pattern was defined as the plant-based dietary pattern (PBDP), mainly including fruits, vegetables, fungi and algae, beans and their products, coarse grains, eggs and their products, and aquatic products. The second dietary pattern was defined as the northern pastoral dietary pattern (NPDP), representing a characteristic diet in the northern pastoral area. This pattern predominantly included beef and mutton, nuts, poultry, fried food, wine, milk tea, milk, and its derivatives. The third dietary pattern was the northern traditional dietary pattern (NTDP), representing a typical traditional diet and mainly including potatoes, wheat flour and its derivatives, rice and related products, pork, salt, and pickled vegetables. Together, these three dietary patterns explained 28.563% of the total variance in the original dietary variables. The adaptability test value of Kaiser-Meyer-Olkin was 0.723, and the Bartlett’s sphericity test was significant (*p* < 0.01), confirming that the dataset was appropriate for PCA (Supplementary Table S2).

### 3.3 Quality evaluation of dietary patterns using Dietary Balance Index-22 scores

For the PBDP, the regression coefficients of LBS and DQD were both less than 0, while the regression coefficient of HBS was greater than 0. As the score of PBDP increased (from Q1 to Q4), LBS and DQD decreased, while HBS increased, indicating a trend toward a more balanced dietary pattern. For the NPDP, the regression coefficients of LBS and DQD were also less than 0, while the regression coefficient of HBS was greater than 0. With increasing adherence to the NPDP (Q1-Q4), LBS and DQD decreased, while HBS increased, further reflecting a tendency toward a more balanced diet. For the NTDP, the regression coefficients for LBS were all less than 0, whereas those for HBS and DQD were greater than 0. As the NTDP factor score increased (Q1-Q4), LBS decreased, while HBS and DQD increased, indicating a shift toward a more unbalanced dietary pattern (Table 3).

**Table 3:** Generalized linear model of dietary quality based on the DBI-22 index.

Dietary pattern	LBS		HBS		DQD	
	*β (95%CI)*	*p*	*β (95%CI)*	*p*	*β (95%CI)*	*p*
PBDP	−4.6 (−4.8,-4.3)	*<0.001*	1.5 (1.3,1.6)	*<0.001*	−3.1 (−3.3,-2.9)	*<0.001*
NPDP	−2.7 (−2.9, −2.4)	*<0.001*	1.6 (1.4,1.8)	*<0.001*	−1.1 (−1.3, −0.8)	*<0.001*
NTDP	−2.1 (−2.3, −1.9)	*<0.001*	2.4 (2.3,2.6)	*<0.001*	0.3 (0.1,0.5)	*<0.001*

The model has been adjusted for age, gender, region, ethnicity, marital status, education, smoking status, and physical activity.

### 3.4 Association between dietary patterns and obesity phenotypes

**Figure 1:**
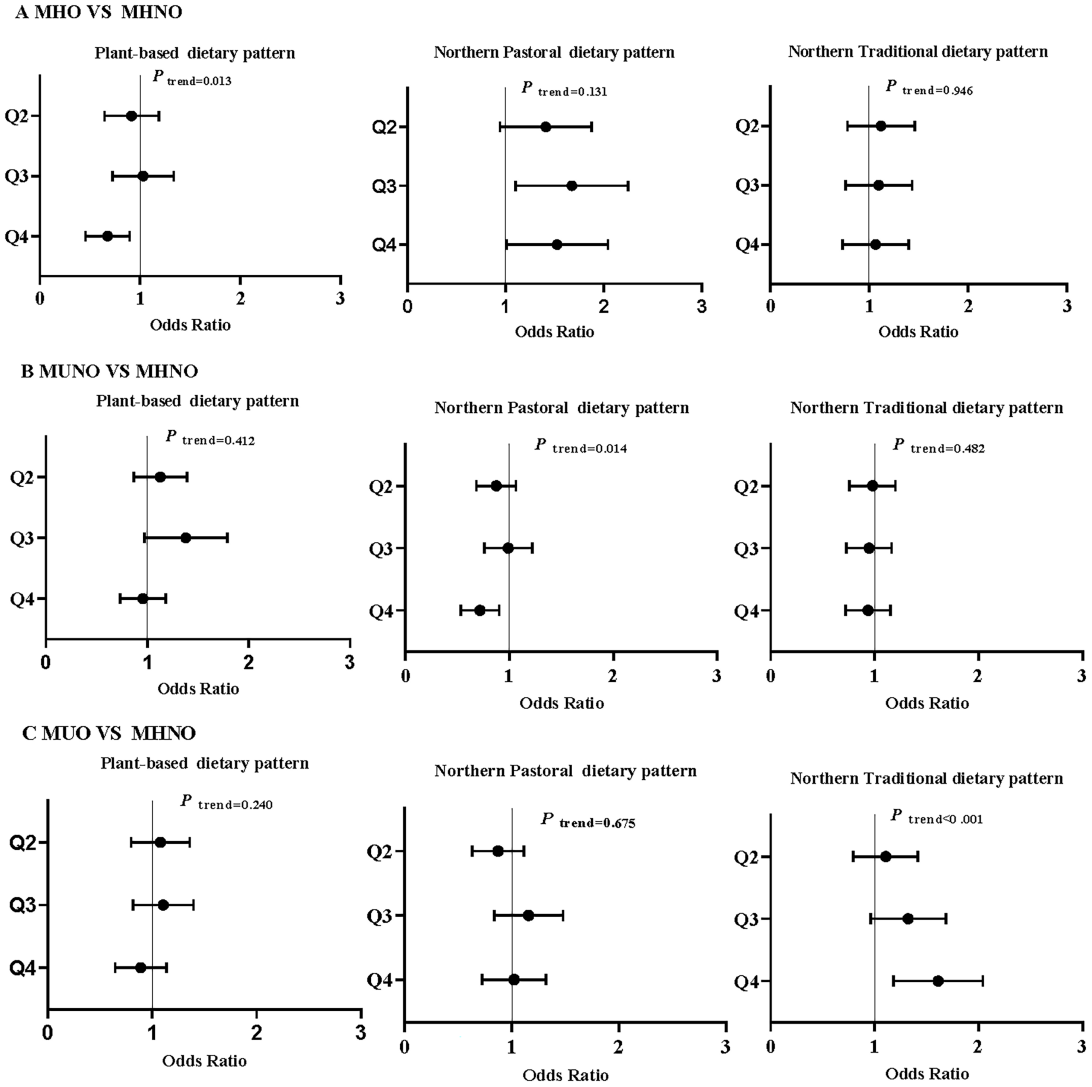
Association between dietary patterns and obesity phenotypes (MHNO as the reference group). Multinomial logistic regression was employed to adjust for age, gender, region, ethnicity, marital status, education, smoking status, and physical activity.Q2-Q4 represent the second to fourth quartile of the diet score of each dietary pattern. The dot represents the estimated odds ratio, and the horizontal line represents the 95% confidence interval.

A multinomial logistic regression model was used to examine the association between dietary patterns and metabolic obesity phenotypes (Figure 1). After adjusting for all aforementioned confounders, three distinct dietary patterns were significantly associated with MHO, MUNO, and MUO phenotypes (using MHNO as the reference group). Individuals with high adherence to the PBDP had a reduced risk of MHO (MHO vs. MHNO, OR 0.65, 95% CI 0.47, 0.91; *P*_trend_ = 0.013). Individuals with high adherence to the NTDP had an increased risk of MUO (MUO vs. MHNO, OR 1.57, 95%CI 1.2, 2.06; *P*_trend_ < 0.001). However, high adherence to the NPDP showed different trends for the three phenotypes. Specifically, individuals with high adherence to the NPDP had increased risk of MHO (MHO vs. MHNO, OR 1.44, 95% CI 1.0, 2.08; *P*_trend_ = 0.131), while showing a reduced risk of MUNO (MUNO vs. MHNO, OR 0.7, 95% CI 0.54, 0.91; *P*_trend_ = 0.014). However, high adherence to the NPDP had no association with MUO.

### 3.5 Subgroup analyses

**Figure 2:**
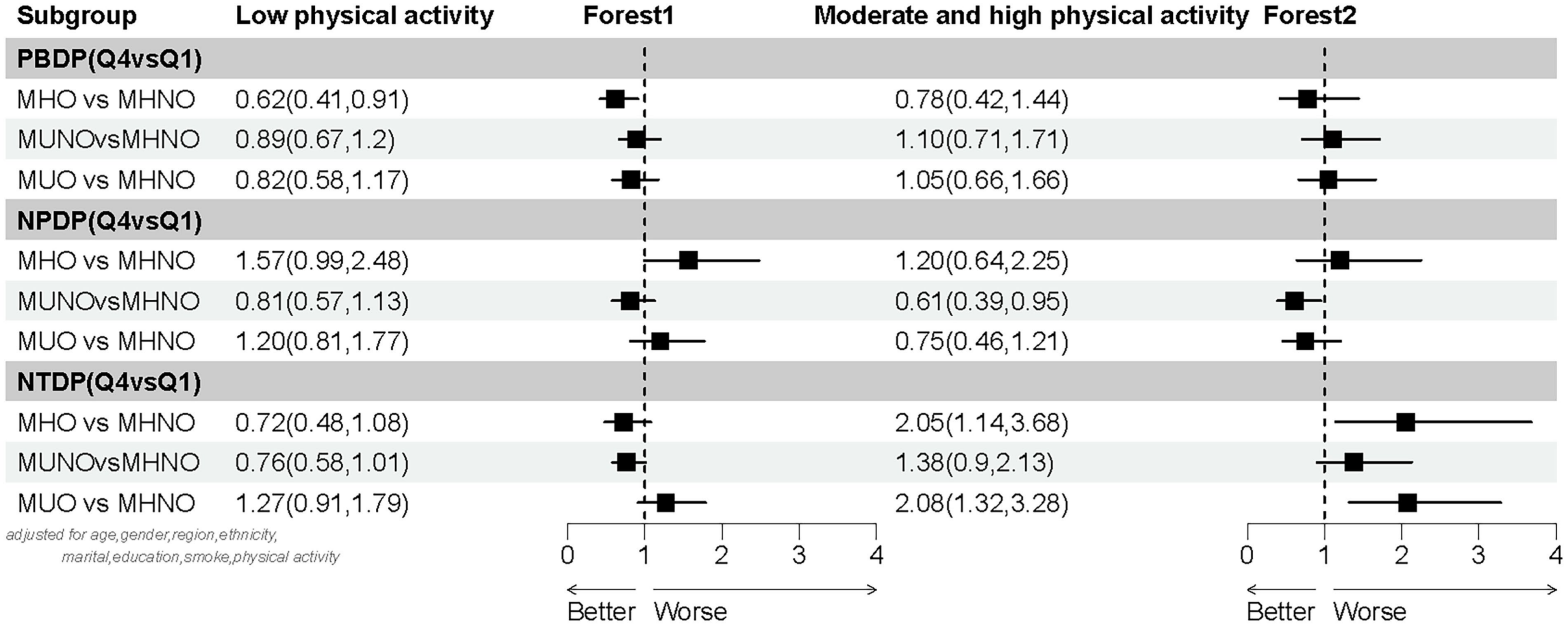
Subgroup analysis based on physical activity (low/moderate and high physical activity).

The subgroup analysis demonstrated that the estimated association between dietary patterns and obesity phenotypes was based on levels of physical activity (Figure 2). After adjusting for age, gender, region, ethnicity, marital status, education, and smoking status, three distinct dietary patterns were significantly associated with the MHO, MUNO, and MUO phenotypes (using MHNO as the reference group), specifically within the low physical activity subgroup. The PBDP was associated with MHO, where individuals in Q4 exhibited a lower risk of MHO compared to those in Q1 (OR 0.62, 95% CI 0.41, 0.91). In the moderate and high physical activity subgroups, the NPDP was associated with MUNO, with individuals in Q4 showing a reduced risk of MUNO relative to Q1 (OR 0.61, 95% CI 0.39, 0.95). Furthermore, the NTDP was associated with both MHO and MUO. Specifically, individuals in Q4 had an elevated risk of MHO (OR 2.05, 95% CI 1.14, 3.68) and MUO (OR 2.08, 95% CI 1.32, 3.28) compared to those in Q1.

## 4 Discussion

The prevalence of different obesity phenotypes was as follows: MHNO, 44.0%; MHO, 11.5%; MUNO, 27.1%; and MUO, 17.4%, indicating that the metabolic abnormalities are more prevalent in obese populations compared to metabolic health, whereas non-obese populations exhibit significantly lower levels of metabolic abnormalities relative to their metabolic health. Similar to our results, a study on Brazilian adults reported that the prevalence of MHNO was 44%, with similar prevalence rates of MUO and MHO phenotypes, both at 23%, whereas the prevalence of MUNO was merely 10% (32). In contrast, a study among adults in southwest China found that 33.28% exhibited the MHO phenotype, 13.80% the MUO phenotype, and 2.95% the metabolically healthy normal weight (MUNW) phenotype (12). The discrepancies in the prevalence of obesity phenotypes across populations may be due to different diet habits and lifestyles (33).

In Inner Mongolia, three distinct dietary patterns were observed: the plant-based dietary pattern (PBDP), the northern pastoral dietary pattern (NPDP), and the northern traditional dietary pattern (NTDP). These three dietary patterns explained 28.563% of the total variance in the original dietary variables. Compared to the findings reported in other studies (34), the dietary patterns examined in this study provided a more robust explanation of dietary habits.

The PBDP was primarily composed of fruits, vegetables, fungi and algae, legumes and their products, whole grains (coarse cereals), eggs and their products, and aquatic products. Compared to the reference group of MHNO, participants who adhered more closely to the PBDP exhibited a reduced risk of MHO. A meta-analysis demonstrated that whole grains (coarse cereals), vegetables, fruits, legumes, and their products, as well as eggs and their derivatives, were associated with a reduced risk of obesity (35). Eggs and aquatic products (such as fish and shellfish) are rich in high-quality animal protein, essential amino acids, omega-3 fatty acids, and various vitamins and minerals, providing significant nutritional value that helps maintain a healthy weight and prevent diabetes and metabolic syndrome (36). However, the relatively low contribution of these two foods to the PBDP may limit the potential for improving metabolic health. Therefore, while the PBDP was effective in reducing obesity risk, it exhibited no significant impact on metabolic abnormalities. PBDP rich in fruits, vegetables, beans, and other foods is a balanced dietary pattern, which helps to achieve optimal health (37). The DBI-22 evaluation results of this study further corroborate that the PBDP constitutes a balanced dietary approach.

The NPDP primarily consisted of beef and mutton, nuts, poultry, fried food, wine, milk tea, as well as milk and its products. Compared to the reference group of MHNO, participants with higher adherence to the NPDP exhibited an increased risk of MHO and a decreased risk of MUNO. Previous studies have demonstrated that high dairy intake was generally linked to a decreased risk of metabolic syndrome components (38). However, inconsistencies exist in the literature regarding its relationship with obesity (38). One possible explanation for the positive correlation between the NPDP and obesity is that a diet characterized by diverse and unrestricted energy intake may promote obesity (39). High energy intake of food (such as beef and mutton, poultry, fried pasta, and alcohol) will increase the risk of obesity (35). In addition, physical activity deficiency is often accompanied by a lower basal metabolic rate, which reduces energy consumption and increases the possibility of obesity (40). Nevertheless, among individuals with moderate or high levels of physical activity, higher adherence to the NPDP has not been observed to increase the risk of obesity but rather to reduce the risk of metabolic abnormalities. These results suggested that moderate and high physical activity may effectively offset the impact of a high-energy diet on body weight by promoting energy balance, thereby helping to prevent obesity to a certain extent (41). Nuts, red meat, milk, and dairy products contain high-quality fat and protein, which can decrease the risk of metabolic syndrome (42). Milk and its products are rich in high-quality protein, calcium, and probiotics, which enhance insulin sensitivity and lipid metabolism, thereby significantly reducing the risk of metabolic abnormalities (43). In addition, the findings reveal that the NPDP tends to be balanced, but the balance is inferior compared to the PBDP. This is because the balanced diet emphasizes not only the diversity of food but also the intake of whole grains, vegetables, and fruits (44), which predominantly characterize the PBDP. Therefore, individuals with higher adherence to the NPDP are advised to improve their weight management through increased physical activity, thereby better maintaining metabolic health.

The intake of high-carbohydrate food was higher in the NTDP. This dietary pattern primarily consisted of potatoes, wheat flour and its products, rice and its products, pork, salt, and pickled vegetables. Compared to the reference group of MHNO, the risk of MUO increased among participants with higher adherence to the NTDP. Overconsumption of high-carbohydrate foods, such as potatoes, wheat, and rice, can lead to weight gain and obesity (45). High carbohydrate intake directly affects the blood glucose load in the human body to reduce insulin sensitivity, which, in turn, affects fat metabolism and ultimately leads to metabolic abnormalities such as dyslipidemia (46). This was a typical traditional dietary pattern similar to that of northern China (47). However, traditional dietary patterns were associated with an increased risk of metabolic syndrome (48). The adverse effects of the NTDP on MUO may stem from dietary imbalances, which are often attributed to food supply limitations and insufficient nutritional awareness in underdeveloped regions (49). Overconsumption of high-salt foods can lead to an imbalance in diet due to the excessive or insufficient intake of certain nutrients, such as sodium ions, potassium ions, fat, and protein (50). The results of this study showed that the NTDP was an unbalanced dietary pattern. Among individuals with moderate and high levels of physical activity, the NTDP was associated with an increased risk of obesity and metabolic abnormalities. This finding could be attributed to the fact that, although moderate and high physical activity helps to control weight (51), poor dietary structure may offset the health benefits of exercise, thereby increasing the risk of obesity and metabolic abnormalities (52). Additionally, insufficient recovery following higher levels of physical activity may lead to elevated inflammatory markers (e.g., C-reactive protein) (53), as well as the accompanying adverse food choices (54). Therefore, it was suggested that individuals with a higher adherence to the NTDP focus on moderating their intake of high-carbohydrate and high-salt foods.

In summary, higher adherence to the balanced NPDP may promote the risk of obesity while helping to prevent metabolic abnormalities, indicating a tendency toward the MHO phenotype. Higher adherence to the balanced PBDP can reduce the risk of obesity. In contrast, higher adherence to the unbalanced NTDP was associated with obesity and metabolic abnormalities, which shows that it urgently needs targeted intervention measures.

Our study had several limitations. First, our analysis relied on self-reported dietary data collected through the FFQ, which is susceptible to recall bias. Second, due to differences in regions, cultures, and socioeconomic status, the dietary patterns may differ across populations, making it challenging to generalize our findings to other groups. Third, despite adjusting for multiple covariates, potential unknown or unmeasured confounding factors may still exist. Future research should include more cohort studies and randomized controlled trials, as well as in-depth analyses of nutrients in dietary patterns and food types, ensuring the identification of the key nutrients that can reduce obesity and metabolic abnormalities.

## 5 Conclusion

We investigated the association between dietary patterns and obesity phenotypes in Inner Mongolia. Specifically, higher adherence to the balanced northern pastoral dietary pattern exhibited dual effects on health outcomes. Meanwhile, higher adherence to the balanced plant-based dietary pattern can reduce the risk of obesity. In contrast, higher adherence to the unbalanced northern traditional dietary pattern may concurrently elevate the risk of both obesity and metabolic abnormalities. These findings hold substantial significance as they reveal the potential role of dietary patterns in the prevention and management of obesity and metabolic abnormalities. However, it is imperative to acknowledge the limitations of this study, and further research is necessary to validate and expand these findings across diverse populations and environmental settings.

## Data Availability

The raw data supporting the conclusions of this article will be made available by the authors, without undue reservation.
