# Supplementary material for: Association between dietary patterns and different obesity phenotypes among Inner Mongolia adults: a cross-sectional study
Source: Front Nutr. 2025 Sep 2;12:1660337. doi: 10.3389/fnut.2025.1660337 (PMC12436458; doi:10.3389/fnut.2025.1660337)
Supplement: Supplementary file 1 [file Table_1.docx]

Supplementary Material

# Supplementary Tables

| **Supplementary Table 1 (Table S1)Metabolic status of different obesity phenotypes** | | | | | |
| --- | --- | --- | --- | --- | --- |
| Characteristics | MHNO | MHO | MUNO | MUO | *P* value |
| Metabolic parameters |  |  |  |  |  |
| BMI (kg/m^2^) | 24±2.3 ^a^ | 25±2 ^b^ | 30.6±2.5 ^c^ | 30.8±2.6 ^b^ | <0.001 |
| SBP (mmHg) | 122.5±17.2 _a_ | 135.6±17.7 _b_ | 126.6±19.4 ^c^ | 138.2±16.7 ^d^ | <0.001 |
| DBP (mmHg) | 78.3±10.6 ^a^ | 86.5±10.9 ^b^ | 80±12.3 ^c^ | 88.6±11.3 ^d^ | <0.001 |
| Glucose(mmol/l) | 4.4±0.7 ^a^ | 5.3±2 ^a^ | 4.4±0.7 ^b^ | 5.2±2 ^b^ | <0.001 |
| Triglycerides(mmol/l) | 1.2±0.6 ^a^ | 2.8±2.3 ^a^ | 1.4±0.7 ^b^ | 2.8±2.2 ^b^ | <0.001 |
| HDL-C(mmol/l) | 1.4±0.3 ^a^ | 1.2±0.3 ^b^ | 1.3±0.2 ^c^ | 1.1±0.2^d^ | <0.001 |
| Other variables |  |  |  |  |  |
| LDL-C(mmol/l) | 3.1±0.7 ^a^ | 3.2±0.8 ^b^ | 3.2±0.8 ^b^ | 3.2±0.8 ^b^ | <0.001 |
| Total cholesterol(mmol/l) | 5.1±1.0 ^a^ | 5.3±1.2 | 5.3±1.0 ^b^ | 5.4±1.1 ^b^ | <0.001 |

Continuous variables are presented as means ± SD, *p*-value is derived from analysis of variance.Different superscript letters in the table represent a *p*-value of <0.05.

| **Supplementary Table 2 (Table S2) Factor loadings of 21 food groups onto three dietary patterns** | | | |
| --- | --- | --- | --- |
| Food groups | PBDP | NPDP | NTDP |
| Fruits | **0.629** | 0.13 | -0.06 |
| Vegetables, mushrooms, and algae | **0.624** | -0.002 | 0.037 |
| Beans and their products | **0.48** | 0.055 | **0.309** |
| Coarse cereals | **0.463** | -0.042 | 0.082 |
| Eggs and their products | **0.393** | 0.025 | 0.204 |
| Aquatic products | **0.393** | **0.308** | 0.029 |
| Beef and mutton | -0.007 | **0.589** | 0.048 |
| Other Drinks | 0.113 | **0.542** | -0.037 |
| Tea, coffee drinks | -0.051 | **0.539** | -0.025 |
| Snack quick dessert nuts | **0.416** | **0.505** | 0.085 |
| Poultry and other meat | **0.378** | **0.44** | 0.108 |
| Fried dough foods | -0.012 | **0.358** | 0.271 |
| Liquor | 0.015 | **0.347** | 0.067 |
| Milk and products thereof | 0.228 | **0.320** | -0.056 |
| Potato | 0.103 | -0.151 | **0.604** |
| Salt and pickle vegetables | 0.086 | -0.009 | **0.492** |
| Wheat and its products | -0.218 | 0.278 | **0.579** |
| Rice and its products | -0.222 | **0.329** | **0.501** |
| Pork | 0.12 | 0.065 | **0.407** |
| Cooking oil | 0.187 | -0.101 | **0.351** |
| Condiments | 0.142 | 0.14 | 0.291 |
| Variance explained (%) | 10.036 | 9.864 | 8.663 |

The extraction method used was principal component analysis, and the rotation method was varimax with orthogonal rotation. Bold indicated factor loadings > 0.3.The cumulative contribution rate of variance was 28.563%; KMO = 0.723.
